# Synthesis, crystal structure, Hirshfeld surface analysis, MEP study and mol­ecular docking of *N*-{3-[(4-meth­oxy­phen­yl)carbamo­yl]phen­yl}-3-nitro­benzamide as a promising inhibitor of hfXa

**DOI:** 10.1107/S2056989020013730

**Published:** 2020-10-20

**Authors:** Rodolfo Moreno-Fuquen, Mario Hurtado-Angulo, Kevin Arango-Daraviña, Gavin Bain, Alan R. Kennedy

**Affiliations:** a Grupo de Cristalografía, Departamento de Química, Universidad del Valle, A.A 25360 Santiago de Cali, Colombia; b WestCHEM. Department of Pure and Applied Chemistry, University of Strathclyde, 295 Cathedral Street, Glasgow G1XL, Scotland

**Keywords:** crystal structure, Hirshfeld surfaces, mol­ecular electrostatic potential, mol­ecular docking

## Abstract

The title compound, C_21_H_17_N_3_O_5_, consists of three rings, *A*, *B* and *C*, linked by amide bonds with the benzene rings *A* and *C* being inclined to the mean plane of the central benzene ring *B* by 2.99 (18) and 4.57 (18)°, respectively. In the crystal, mol­ecules are linked *via* N—H⋯O and C—H⋯O hydrogen bonds, forming fused *R* ^2^_2_(18), *R* ^3^_4_(30), *R* ^4^_4_(38) rings running along [0] and *R* ^3^_3_(37) and *R* ^3^_3_(15) rings along [001]. Hirshfeld analysis was undertaken to study the inter­molecular contacts in the crystal, showing that the most significant contacts are H⋯O/O⋯H (30.5%), H⋯C/C⋯H (28.2%) and H⋯H (29.0%). Two zones with positive (50.98 and 42.92 kcal mol^−1^) potentials and two zones with negative (−42.22 and −34.63 kcal mol^−1^) potentials promote the N—H⋯O inter­actions in the crystal. An evaluation of the mol­ecular coupling of the title compound and the protein with enzymatic properties known as human coagulation factor Xa (hfXa) showed the potential for coupling in three arrangements with a similar minimum binding energy, which differs by approximately 3 kcal mol^−1^ from the value for the mol­ecule Apixaban, which was used as a positive control inhibitor. This suggests the title compound exhibits inhibitory activity.

## Abstract 2

The structure of the title compound is stabilized by the presence of N—H⋯O and C—H⋯O hydrogen bonds. Other inter­actions such as C—H⋯π are also important in the analysis of the Hirshfeld surface. Mol­ecular docking studies show this compound to be a potential anti­coagulant agent.

## Chemical context   

The synthesis of new compounds derived from amino­benzamides has generated a growing inter­est in the search for mol­ecular systems that can have different physical, chemical or biological properties. Some amino­benzamide derivatives present high efficiencies in their non-linear optical properties (Prasad *et al.*, 2011 ▸). Exceptional insecticidal activity against lepidopterous insects has been shown by some anthranilic di­amides (Lahm *et al.*, 2005 ▸) and benzene dicarboxamide derivatives (Tohnishi *et al.*, 2005 ▸; Lahm *et al.*, 2007 ▸). Other substituted benzamides are used as medications for patients with schizophrenia (Racagni *et al.*, 2004 ▸). Some benzamide compounds are used in the area of neurology for their powerful neuroprotective effects (Hirata *et al.*, 2018 ▸). Other di­amide compounds analogous to the product obtained in this work have been tested as potential options for anti­thrombotic treatments by acting as direct inhibitors of coagulation factor Xa (Xing *et al.*, 2018 ▸; Lee *et al.*, 2017 ▸). This study was undertaken with the aim of providing new compounds with possible pharmaceutical applications. The results of the synthesis, crystal-structure determination by single-crystal X-ray diffraction, supra­molecular analysis and an evaluation of mol­ecular coupling on human factor Xa of the title compound, (I), a new di­amide derived from 3-amino­benzamide, is presented here.
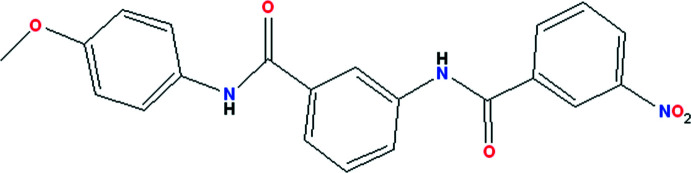


## Structural commentary   

**Figure 1:**
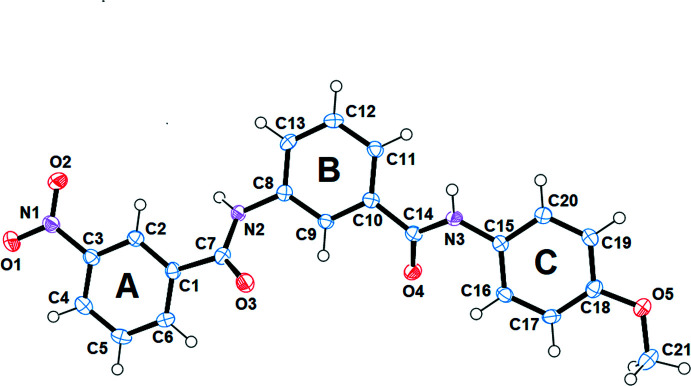
The mol­ecular structure of (I), showing the atom labeling and displacement ellipsoids drawn at the 50% probability level.

In the title compound, Fig. 1 ▸, the mean planes of rings *A* (C1–C6; r.m.s deviation = 0.0127 Å) and *C* (C15–C20; r.m.s deviation = 0.0086 Å) form dihedral angles of of 2.99 (18) and 4.57 (18)°, respectively, with the mean plane of the central ring *B* (C8–C13; r.m.s deviation = 0.0072 Å). In turn, the amide groups C8/N2/C7(O3)/C1 (r.m.s deviation = 0.0081 Å) and C15/N3/C14(O4)/C10 (r.m.s deviation = 0.0101 Å), which link the rings, are inclined by 37.72 (15) and 29.35 (16)°, respectively, to ring *B*. The nitro group forms an angle of 5.3 (2)° with ring *A* while the meth­oxy group is approximately coplanar with ring *C*, forming an angle of 1.1 (5)°. The mol­ecule is formed by three main planes resulting from the planes that form the rings with the *B* ring resembling a fallen step between *A* and *C*. All bond lengths (Allen *et al.*, 1987 ▸) and bond angles are within normal ranges.

## Supra­molecular features   

**Figure 2:**
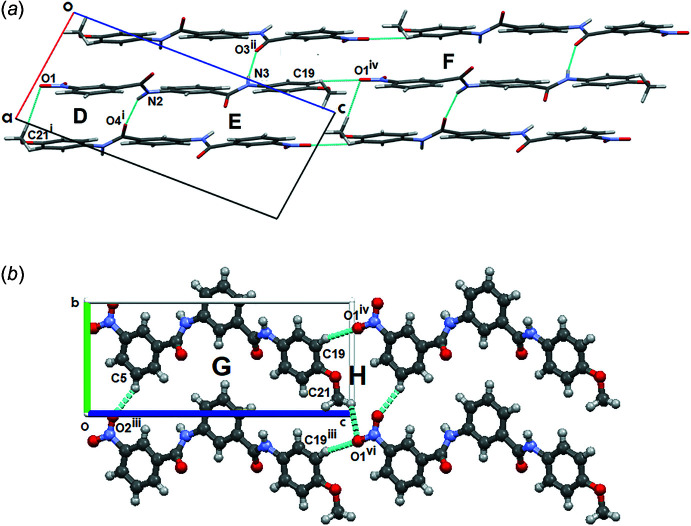
(*a*) Partial crystal structure of (I), showing the formation of **D** = 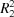
(18), ***E*** = 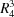
(30) and **F** = 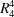
(38) fused rings running along [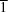
0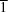] and (*b*) **G** = 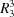
(37) and **H** = 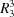
(15) fused rings. These fused rings run along [001]. Symmetry code: (vi) *x* − 1, *y* − 1, *z* + 1.

The crystalline packing in compound (I) is mainly regulated by the presence of N—H⋯O and C—H⋯O hydrogen bonds. The N2—H2*N*⋯O4^i^ and N3—H3*N*⋯O3^ii^ inter­actions [symmetry codes: (i) −*x* + 1, *y* + 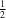, −*z* + 1; (ii) −*x*, *y* + 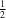, −*z* + 1] form fused 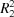
(18), 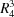
(30) and 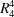
(38) rings (Etter, 1990 ▸) running along [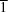
0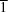] (see Table 1 ▸ and Fig. 2 ▸ *a*). In turn, playing the role of complementary inter­actions in crystal growth, other C—H⋯O-type inter­actions [C19—H19⋯O1^iv^ and C21—H21*C*⋯O1^v^; symmetry codes: (iv) *x* − 1, *y*, *z* + 1; (v) −*x* + 1, *y* − 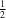, −*z* + 1] link the mol­ecules at their ends, ensuring their stability in the growth process. Together with the N—H⋯O inter­actions, they contribute to the formation of additional fused 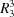
(37) and 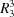
(15) rings, which run along [001] (see Table 1 ▸, Fig. 2 ▸ *b*). Weak C21—H21*B*⋯*Cg*1 inter­actions occur, where *Cg*1 is the centroid of the C1–C6 benzene ring, with a H21*B*⋯*Cg*1 distance of 2.83 Å.

## Hirshfeld surface analysis   

**Figure 3:**
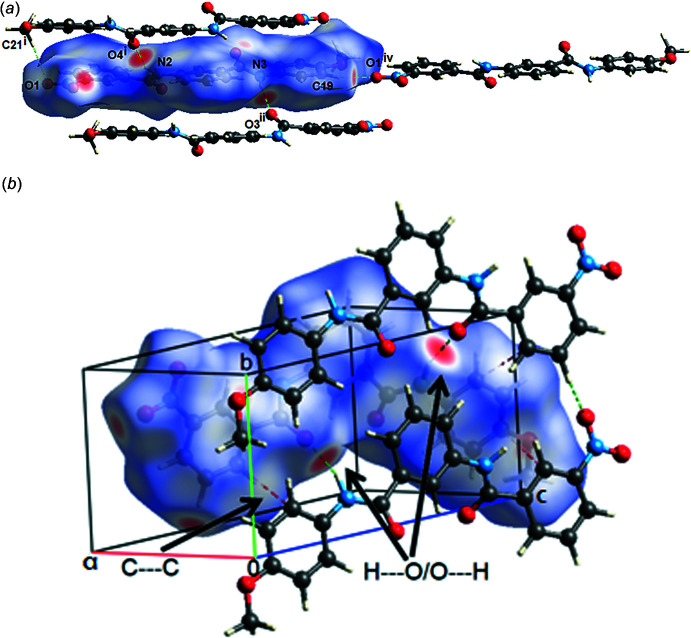
(*a*) A view of the Hirshfeld surface of (I) mapped over *d*_norm_ emphasizing the N—H⋯O and C—H⋯O inter­molecular inter­actions and (*b*) showing the H⋯O/O⋯H and C⋯C (π–π) inter­actions. Symmetry codes: (i) −*x* + 1, *y* + 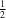, −*z* + 1; (ii) −*x*, *y* + 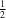, −*z* + 1; (iii) *x* − 1, *y*, *z* + 1.

**Figure 4:**
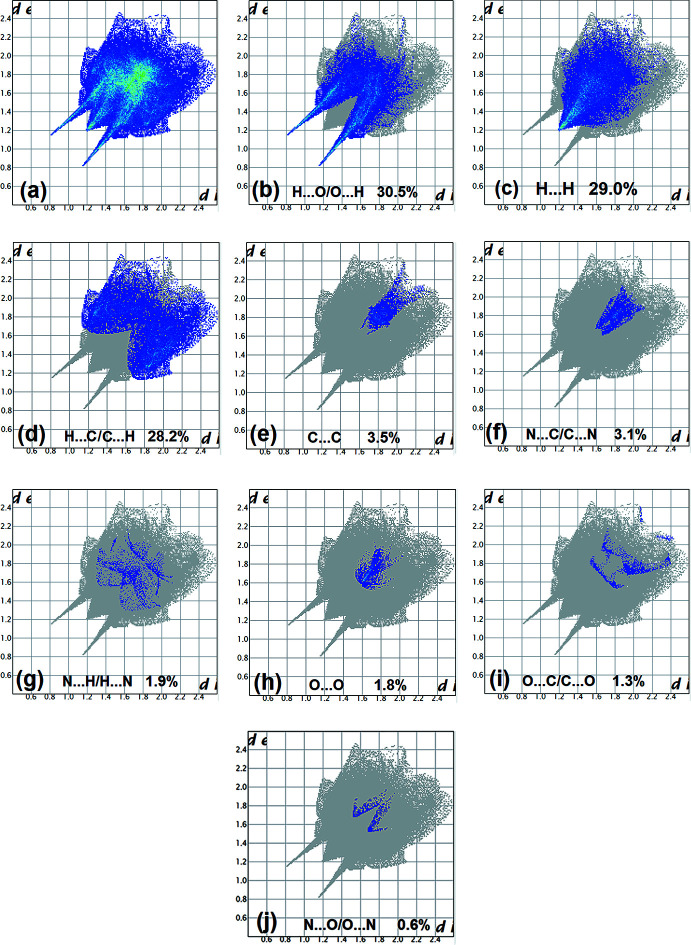
Two-dimensional fingerprints plots for the title compound, showing (*a*) all inter­actions, (*b*) H⋯O/O⋯H, (*c*) H⋯H, (*d*) H⋯C/C⋯H, (*e*) C⋯C, (*f*) N⋯C/C⋯N, (*g*) H⋯N/N⋯H, (*h*) O⋯O, (*i*) O⋯C/C⋯O, and (*j*) N⋯O/O⋯N inter­actions. The *d*_i_ and *d*_e_ values are the closest inter­nal and external distances (in Å) of given points on the Hirshfeld surface.

Inter­molecular inter­actions, including hydrogen-bond inter­actions, are essential in reinforcing the stability of the supra­molecular structure. This behavior can be analyzed through the study of Hirshfeld surfaces (HS) using the *CrystalExplorer* program (Spackman & Jayatilaka, 2009 ▸), which allows the visualization of the inter­actions within the crystal structure, including N⋯H and C⋯H inter­actions. To examine in depth the strength and capacity of hydrogen bonds and other inter­molecular contacts, one of the Hirshfeld surface analysis tools, the normalized contact distance, *d*_norm_, has been used (Turner *et al.*, 2017 ▸). The results show that the most important contributions to the crystal packing are due to the N—H⋯O and C—H⋯O hydrogen bonds. This is evidenced by the more intense red spots on the HS for (I) (see Fig. 3 ▸ *a*,*b*). The overall two-dimensional fingerprint plot (Fig. 4 ▸ *a*), and those delineated into by H⋯O/O⋯H, H⋯H, H⋯C/C⋯H, C⋯C, N⋯C/C⋯N, H⋯N/N⋯H, O⋯O, O⋯C/C⋯O, and N⋯O/O⋯N contacts are illustrated in Fig. 4 ▸ *b*–*j*, respectively, together with their relative contributions to the Hirshfeld surface. The most important inter­action corresponds to H⋯O/O⋯H contributing 30.5% to the overall crystal packing, as shown in Fig. 4 ▸ *b*. The pair of spikes in the fingerprint plot have a symmetrical distribution of high-density points with the tip at *d*_e_ + *d*_i_ = 1.98 Å. The H⋯H inter­actions, shown in Fig. 4 ▸ *c*, contribute 29.0% to the total crystal packing and appear as widely dispersed points of high density due to the large hydrogen-atom content of the mol­ecule with the rounded tip at *d*_e_ + *d*_i_ = 1.20 Å. The presence of C—H⋯π inter­actions is shown as a pair of characteristic wings on the fingerprint plot (Fig. 4 ▸ *d*), delineated into H⋯C/C⋯H contacts (28.2% contribution to the HS) having the tip at *d*_e_ + *d*_i_ = 2.84 Å. These results reveal the importance of H-atom contacts in establishing the packing. The large number of H⋯H, H⋯C/C⋯H and H⋯O/O⋯H inter­actions suggest that van der Waals inter­actions and hydrogen bonding play major roles in the crystal packing (Hathwar *et al.*, 2015 ▸).

## Mol­ecular Electrostatic Potential (MEP)   

**Figure 5:**
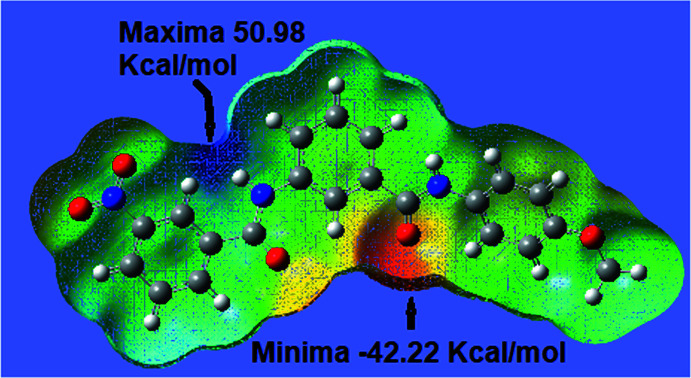
Three-dimensional representation of the electrostatic potential around a mol­ecule of (I).

A study of the mol­ecular electrostatic potential (MEP) of (I) using the *Gauss09W* (Frisch *et al.*, 2009 ▸) and *Gauss View 5.0* programs, at the DFT/B3LYP/6-31G (d, p) level of theory, to obtain a qualitative analysis and the *Multiwfn 3.6* program for a qu­anti­tative analysis (Lu & Chen, 2012 ▸) of the surface was undertaken. On the potential surface, marked in dark blue, positive regions of low electron density that can suffer nucleophilic attacks in a chemical reaction or can have inter­actions with nucleophiles in the process of crystalline growth are shown (see Fig. 5 ▸). Two zones show positive potentials of 50.98 and 42.92 kcal mol^−1^ in regions that follow the directions of the N—H bonds above and below the plane through the rings of the mol­ecule and which promote the formation of the N2—H2*N*⋯O4^i^ and N3—H3*N*⋯O3^ii^ hydrogen bonds throughout the crystal. In turn, other areas with high density, being close to the carbonyl oxygen atoms and shown in a reddish color, show values of −42.22 and −34.63 kcal mol^−1^. Thus, in the first stage of crystal formation, these low and high electronic density regions are inter­twined to promote the formation of rings and chains of mol­ecules along the crystal. The other areas with lower values in the regions will be accommodated to enable the formation of other weaker bonds, C5—H5⋯O2^iii^, C19—H19⋯1^iv^ and C21—H21*C*⋯O1^v^, representing the growth characteristics of each crystal.

## Mol­ecular Docking Evaluation   

**Figure 6:**
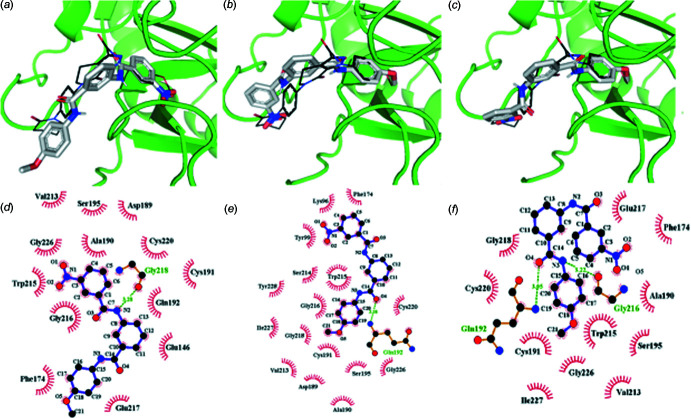
Models of mol­ecular coupling for inhibition of hfXa by compound (I) in its poses: (*a*) and (*d*) pose 1; (*b*) and (*e*) pose 2, and (*c*) and (*f*) pose 3. Dashed lines indicate hydrogen bonds. Carbon atoms are in black, nitro­gen in blue and oxygen in red.

One of the newest synthetic direct-acting compounds to be licensed for the treatment of therapeutic anti­coagulation is Apixaban. To evaluate its potential as an anti­coagulant agent, a mol­ecular docking study of (I) as a ligand and the human coagulation factor hfXa as the receptor protein was performed. For the mol­ecular coupling calculations, the free software *Autodock Vina 4.2.6* (Trott & Olson, 2010 ▸) was used, and as a positive control ligand in the active site, the mol­ecule Apixaban (PDB code 2p16; Pinto *et al.*, 2007 ▸), one of the most important substrates currently accepted by the US Food and Drug Administration (FDA) for anti­thrombotic treatments (Agnelli *et al.*, 2013 ▸) was used. The Apixaban mol­ecule was also used as a model for the validation and verification of the parameters of the mol­ecular coupling calculations performed. The bonding energy obtained for the new inhibitor was −7.7 and −10.7 kcal mol^−1^ for the control ligand (Table 2 ▸). Compound (I), as a ligand at the active site of hfXa, presents one hydrogen bond of medium strength between the local N2 atom and the amino acid residue Gly218. However, all hydro­phobic residue contacts of the latter in its more stable configuration are also present in the positive control ligand with the exception of Glu217. The results of the mol­ecular coupling calculations show that ligand (I) has two equivalent energy configurations, poses 2 and 3 (see Table 3 ▸), which differ energetically from pose 1 by only 0.1 and 0.3 kcal mol^−1^, respectively. Thus, pose 3 presents a better 3D conformational arrangement at the active site, this pose being much more similar to that of the control ligand (see Fig. 6 ▸). This behavior implies that two of the three hydrogen bonds presented by the control ligand in the active site Gly216 and Gln192, plus all hydro­phobic contacts of ligand (I) in its pose 3, coincide with those presented by the control ligand. For this reason, the difference in binding energy between (I) and the control ligand at the active site is due to the chemical difference between the functional groups present in each structure. The mol­ecular coupling images were generated using the programs *PyMOL* (Rigsby & Parker, 2016 ▸) and *Ligplot+* (Laskowski & Swindells, 2011 ▸).

## Database survey   

A search of the Cambridge Structural Database (CSD, version 5.41, November 2019 update; Groom *et al.*, 2016 ▸) using [3-(benzoyl-λ^2^-azan­yl)phen­yl](phenyl-λ^2^-azan­yl)methanone as the main skeleton gave 76 hits. Seven structures containing the [3-(benzoyl-λ^2^-azan­yl)phen­yl](phenyl-λ^2^-azan­yl)methanone framework with nitro and meth­oxy groups as substituents similar to the title compound were found, *viz*., *N*-{3-[*N*′-(2-meth­oxy­phen­yl)carbamo­yl]-5-methyl-2-meth­oxy­phen­yl}-2-meth­oxy-5-methyl-3-nitro­benzamide (HEXXOY; Yi *et al.*, 2007 ▸), *N*-{3-[*N*′-(2-meth­oxy­phen­yl)carbamo­yl]-5-methyl-2-meth­oxy­phen­yl}-2-hy­droxy-5-methyl-3-nitro­benzamide (HEXYAL; Yi *et al.*, 2007 ▸), methyl 3-({2-hy­droxy-3-[(2-meth­oxy­benzo­yl)amino]­benzo­yl}amino)-2-meth­oxy­benzoate (POQMEP; Liu *et al.*, 2014 ▸), *catena*-[[μ-methyl 3-({2-oxy-3-[(2-meth­oxy-3-nitro­benzo­yl)amino]­benzo­yl}amino)-2-meth­oxy­benzoate]aqua­sodium methanol solvate] (POQMIT; Liu *et al.*, 2014 ▸), methyl 2-meth­oxy-3-({2-meth­oxy-3-[(2-meth­oxy-3-nitro­benzo­yl)amino]­benzo­yl}amino)­benzoate (YUXMIO; Yan *et al.*, 2010 ▸), methyl 3-({3-[(2,5-dimeth­oxy-3-nitro­benzo­yl)amino]-2-meth­oxy­benzo­yl}amino)-2,5-di­meth­oxy­benzoate (YUXNEL; Yan *et al.*, 2010 ▸), and methyl 3-({3-[(2,5-dimeth­oxy-3-nitro­benzo­yl)amino]-2,5-di­meth­oxy­benzo­yl}amino)-2,5-di­meth­oxy­benzoate (YUXNOV; Yan *et al.*, 2010 ▸). The specific characteristics of these di­amide systems depend on the presence of the diverse substituents and their position in the rings in such a way that the planarity in the system is maintained between two rings and the third ring is rotated with respect to the first two. In this way, the compounds POQMEP, POQMIT and HEXXOY present a relatively small deviation from planarity, while the systems YUXNOV, YUXNEL, HEXYAL, YUXMIO progressively lose this planarity, ending up with quite large dihedral angles between the planes.

## Synthesis and crystallization   

3-Amino-*N*-(4-meth­oxy­phen­yl)benzamide (ii) (50.5 mg, 0.129 mmol), previously synthesized, was subjected to an acyl­ation reaction in 3-nitro­benzoyl chloride (*a*) (50.5 mg, 0.272 mmol) under chloro­form reflux conditions to obtain compound (I) [yield 44.7 mg (0.114 mmol), 88.4%; m.p. 502 (1) K].
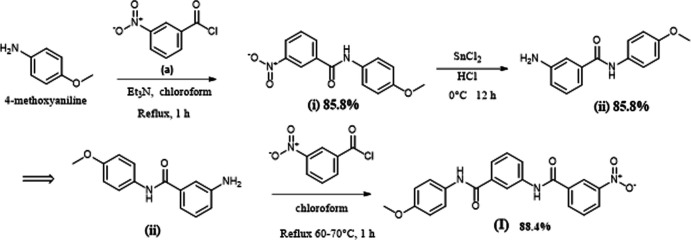


FT–IR (ATR): υ (cm^−1^) = 3338, 3285 (N—H), 3076, 2969, 1650, 1637 (C=O), 1585 (C=C), 1526, 1510, 1434, 1353 (NO_2_), 1316, 1301, 1241, 1137, 1026, 898, 829, 819, 721, 683 cm^−1^. The UV–Vis spectrum for (I) (60 µ*M*) was obtained in DMF with a maximum of 273 nm (0.62 absorption). MS (IE, 40 eV), *m*/*z* [*M* ^+^] calculated for C_21_H_17_N_3_O_5_^+^: 391.12 (100%) and 392.12 (23.1%); found: 391.10 and 392.10 (25.1%).

## Refinement   

Crystal data, data collection and structure refinement details are summarized in Table 4 ▸. All the H atoms were found in a difference-Fourier map and were positioned at geometrically idealized positions, C—H = 0.95 Å (ring), N—H = 0.88 Å and C—H = 0.98 Å (meth­yl) and were refined using a riding-model approximation, with *U*_iso_(H) = 1.2*U*_eq_(parent atom) or 1.5*U*_eq_(C). After refinement, ROTAX suggested twinning by a 180° about [100]. This law was used to generate an hklf5 format reflection file. Refinement against this file improved *R* factors and residual *Q* peaks·BASF refined to 0.090 (4)

## Supplementary Material

Crystal structure: contains datablock(s) I. DOI: 10.1107/S2056989020013730/dj2013sup1.cif

Structure factors: contains datablock(s) I. DOI: 10.1107/S2056989020013730/dj2013Isup2.hkl

Synthesis and characterization of (I). DOI: 10.1107/S2056989020013730/dj2013sup3.pdf

Supporting information file. DOI: 10.1107/S2056989020013730/dj2013Isup4.cml

CCDC reference: 2016019

Additional supporting information:  crystallographic information; 3D view; checkCIF report

## Figures and Tables

**Table 1:** Hydrogen-bond geometry (Å, °)

*D*—H⋯*A*	*D*—H	H⋯*A*	*D*⋯*A*	*D*—H⋯*A*
N2—H2*N*⋯O4^i^	0.88	2.09	2.908 (4)	155
N3—H3*N*⋯O3^ii^	0.88	2.21	3.056 (4)	163
C5—H5⋯O2^iii^	0.95	2.28	3.093 (5)	143
C19—H19⋯O1^iv^	0.95	2.44	3.365 (5)	164
C21—H21*C*⋯O1^v^	0.98	2.64	3.490 (5)	145

Symmetry codes: (i) 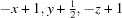; (ii) 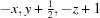; (iii) 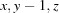; (iv) 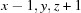; (v) 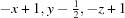.

**Table 2:** Mol­ecular coupling calculations (kcal mol^−1^) for the active hfXa site, the title compound, and the Apixaban mol­ecule

Compound	Bond energy*^*a*^*	No. of hydrogen bonds*^*b*^*	Hydrogen-bonding inter­action residues*^*b*^*	van der Waals inter­action residues*^*b*^*
(I)	−7.7	1	Gly218	Trp215, Cys191, Ser195, Gln192, Cys220, Gly218, Ala190, Gly216, Gly 226, Asp189, Val213, Phe174, Glu217, Glu 146
Apixaban*^*c*^*	−10.7	3	Gly216, Gln192, Glu146	Cys220, Tyr99, Glu97, Phe174, Thr98, Val213, Trp215, Gly226, Cys191, Asp189, Ser195, Ile227, Ala190, Gly218, Arg143

Notes: (*a*) The binding energy indicates the affinity and binding capacity for the active site of the enzyme protein fXa; (*b*) The number of hydrogen bonds and all amino acid residues involved in the enzyme-inhibitor complex were determined using *AutoDock 4.2.6* (Trott & Olson, 2010 ▸); (*c*) The Apixaban mol­ecule was used as a positive control ligand for the active site.

**Table 3:** Equivalent poses of compound (I) in the active hfXa site, as a result of mol­ecular coupling calculations (kcal mol^−1^)

Compound	Bond energy*^*a*^*	No. of hydrogen bonds*^*b*^*	Hydrogen-bonding inter­action residues*^*b*^*	van der Waals inter­action residues*^*b*^*
(I) (pose 1)	−7.7	1	Gly218	Trp215, Cys191, Ser195, Gln192, Cys220, Gly218, Ala190, Gly216, Gly 226, Asp189, Val213, Phe174, Glu217, Glu 146
(I) (pose 2)	−7.6	1	Gly192	Phe174, Tyr99, Val213, Gly226, Trp215, Ser195, Asp189, Ala190, Gly218, Gly216, Cys191, Gln192, Ile 227, Lys96
(I) (pose 3)	−7.4	2	Gly 216, Gln192	Cys220, Glu217, Phe174, Val213, Gly226, Cys191, Ser195, Ala190, Trp215, Gly218, Ile227

Notes: (*a*) The binding energy indicates the affinity and binding capacity for the active site of the enzyme protein fXa; (*b*) The number of hydrogen bonds and all amino acid residues involved in the enzyme-inhibitor complex were determined using *AutoDock 4.2.6* (Trott & Olson, 2010 ▸); (*c*) The Apixaban mol­ecule was used as a positive control ligand for the active site.

**Table 4:** Experimental details

Crystal data	
Chemical formula	C_21_H_17_N_3_O_5_
*M*_r_	391.37
Crystal system, space group	Monoclinic, *P*2_1_
Temperature (K)	123
*a*, *b*, *c* (Å)	7.2948 (3), 7.1242 (3), 16.8297 (7)
β (°)	97.644 (3)
*V* (Å^3^)	866.86 (6)
*Z*	2
Radiation type	Cu *K*α
μ (mm^−1^)	0.91
Crystal size (mm)	0.50 × 0.40 × 0.18

Data collection	
Diffractometer	Oxford Diffraction Gemini S
Absorption correction	Multi-scan
*T*_min_, *T*_max_	0.603, 1.000
No. of measured, independent and observed [*I* > 2σ(*I*)] reflections	3172, 3172, 2982
*R*_int_	0.048
(sin θ/λ)_max_ (Å^−1^)	0.620

Refinement	
*R*[*F* ^2^ > 2σ(*F* ^2^)], *wR*(*F* ^2^), *S*	0.047, 0.126, 1.06
No. of reflections	3172
No. of parameters	263
No. of restraints	3
H-atom treatment	H-atom parameters constrained
Δρ_max_, Δρ_min_ (e Å^−3^)	0.23, −0.30
Absolute structure	Flack *x* determined using 524 quotients [(*I* ^+^)−(*I* ^−^)]/[(*I* ^+^)+(*I* ^−^)] (Parsons *et al.*, 2013 ▸)
Absolute structure parameter	0.2 (3)

Computer programs: *CrysAlis PRO* (Agilent, 2014 ▸), *SIR92* (Altomare *et al.*, 1994 ▸), *SHELXL2014/7* (Sheldrick, 2015 ▸), *Mercury* (Macrae *et al.*, 2020 ▸) and *ORTEP-3 for Windows* (Farrugia, 2012 ▸).

## supplementary crystallographic information

### Crystal data

**Table:**

C_21_H_17_N_3_O_5_	*D*_x_ = 1.499 Mg m^−^^3^
*M**_r_* = 391.37	Melting point: 502(1) K
Monoclinic, *P*2_1_	Cu *K*α radiation, λ = 1.54184 Å
*a* = 7.2948 (3) Å	Cell parameters from 3172 reflections
*b* = 7.1242 (3) Å	θ = 5.3–73.1°
*c* = 16.8297 (7) Å	µ = 0.91 mm^−^^1^
β = 97.644 (3)°	*T* = 123 K
*V* = 866.86 (6) Å^3^	Fragment cut from large slab, pale yellow
*Z* = 2	0.50 × 0.40 × 0.18 mm
*F*(000) = 408	

### Data collection

**Table:**

Oxford Diffraction Gemini S diffractometer	2982 reflections with *I* > 2σ(*I*)
Radiation source: sealed tube	*R*_int_ = 0.048
ω scans	θ_max_ = 73.1°, θ_min_ = 5.3°
Absorption correction: multi-scan	*h* = −8→6
*T*_min_ = 0.603, *T*_max_ = 1.000	*k* = −8→8
3172 measured reflections	*l* = −20→20
3172 independent reflections	

### Refinement

**Table:**

Refinement on *F*^2^	Hydrogen site location: inferred from neighbouring sites
Least-squares matrix: full	H-atom parameters constrained
*R*[*F*^2^ > 2σ(*F*^2^)] = 0.047	*w* = 1/[σ^2^(*F*_o_^2^) + (0.066*P*)^2^ + 0.3205*P*] where *P* = (*F*_o_^2^ + 2*F*_c_^2^)/3
*wR*(*F*^2^) = 0.126	(Δ/σ)_max_ < 0.001
*S* = 1.06	Δρ_max_ = 0.23 e Å^−^^3^
3172 reflections	Δρ_min_ = −0.30 e Å^−^^3^
263 parameters	Absolute structure: Flack *x* determined using 524 quotients [(*I*^+^)-(*I*^-^)]/[(*I*^+^)+(*I*^-^)] (Parsons *et al.*, 2013)
3 restraints	Absolute structure parameter: 0.2 (3)

### Special details

**Table:**

Experimental. CrysAlisPro, Agilent Technologies, Version 1.171.37.35 (release 13-08-2014 CrysAlis171 .NET) (compiled Aug 13 2014,18:06:01) Empirical absorption correction using spherical harmonics, implemented in SCALE3 ABSPACK scaling algorithm.
Geometry. All esds (except the esd in the dihedral angle between two l.s. planes) are estimated using the full covariance matrix. The cell esds are taken into account individually in the estimation of esds in distances, angles and torsion angles; correlations between esds in cell parameters are only used when they are defined by crystal symmetry. An approximate (isotropic) treatment of cell esds is used for estimating esds involving l.s. planes.
Refinement. Refined as a two-component twinAfter refinement ROTAX suggested twinning by a 180 rotation about direct 1 0 0.This law was used to generate a hklf5 formated reflection file.Refinement against this file improved R factors and residual Q peaks.BASF refined to 0.090 (4)

### Fractional atomic coordinates and isotropic or equivalent isotropic displacement parameters (Å^2^)

**Table:**

	*x*	*y*	*z*	*U*_iso_*/*U*_eq_	
O1	0.6697 (5)	0.7564 (5)	0.02367 (18)	0.0376 (8)	
O2	0.5804 (5)	0.9765 (5)	0.09701 (19)	0.0350 (7)	
O3	0.2455 (4)	0.5231 (5)	0.35551 (16)	0.0282 (6)	
O4	0.2775 (4)	0.5306 (4)	0.64175 (16)	0.0263 (6)	
O5	−0.1675 (4)	0.3265 (4)	0.93736 (17)	0.0277 (7)	
N1	0.6116 (4)	0.8119 (5)	0.0852 (2)	0.0246 (7)	
N2	0.3987 (4)	0.8014 (5)	0.37262 (18)	0.0197 (7)	
H2N	0.4792	0.8744	0.3531	0.024*	
N3	0.0451 (4)	0.7137 (5)	0.67641 (19)	0.0198 (7)	
H3N	−0.0181	0.8174	0.6646	0.024*	
C1	0.4599 (5)	0.5945 (6)	0.2656 (2)	0.0200 (8)	
C2	0.5006 (5)	0.7309 (6)	0.2117 (2)	0.0201 (7)	
H2	0.4757	0.8598	0.2199	0.024*	
C3	0.5793 (5)	0.6713 (6)	0.1453 (2)	0.0214 (8)	
C4	0.6237 (5)	0.4862 (7)	0.1322 (2)	0.0249 (8)	
H4	0.6775	0.4510	0.0860	0.030*	
C5	0.5880 (5)	0.3544 (6)	0.1879 (2)	0.0263 (9)	
H5	0.6220	0.2271	0.1817	0.032*	
C6	0.5021 (5)	0.4077 (6)	0.2532 (2)	0.0223 (8)	
H6	0.4718	0.3150	0.2899	0.027*	
C7	0.3569 (5)	0.6363 (6)	0.3358 (2)	0.0212 (8)	
C8	0.3212 (5)	0.8658 (6)	0.4411 (2)	0.0187 (8)	
C9	0.2817 (5)	0.7434 (6)	0.5012 (2)	0.0186 (7)	
H9	0.3051	0.6129	0.4968	0.022*	
C10	0.2080 (5)	0.8120 (6)	0.5675 (2)	0.0190 (8)	
C11	0.1753 (5)	1.0041 (6)	0.5751 (2)	0.0207 (8)	
H11	0.1239	1.0508	0.6202	0.025*	
C12	0.2191 (5)	1.1265 (6)	0.5156 (2)	0.0229 (8)	
H12	0.1986	1.2574	0.5207	0.028*	
C13	0.2922 (5)	1.0588 (6)	0.4492 (2)	0.0213 (8)	
H13	0.3225	1.1433	0.4093	0.026*	
C14	0.1800 (5)	0.6713 (6)	0.6309 (2)	0.0199 (8)	
C15	−0.0029 (5)	0.6049 (6)	0.7415 (2)	0.0185 (8)	
C16	0.0253 (5)	0.4121 (6)	0.7483 (2)	0.0217 (8)	
H16	0.0805	0.3463	0.7085	0.026*	
C17	−0.0274 (5)	0.3165 (6)	0.8134 (2)	0.0217 (8)	
H17	−0.0067	0.1850	0.8181	0.026*	
C18	−0.1096 (5)	0.4096 (6)	0.8715 (2)	0.0218 (8)	
C19	−0.1431 (6)	0.6016 (6)	0.8636 (2)	0.0249 (9)	
H19	−0.2038	0.6659	0.9021	0.030*	
C20	−0.0880 (5)	0.6982 (6)	0.7997 (2)	0.0236 (9)	
H20	−0.1082	0.8297	0.7954	0.028*	
C21	−0.1360 (6)	0.1300 (7)	0.9478 (3)	0.0312 (10)	
H21A	−0.1834	0.0882	0.9967	0.047*	
H21B	−0.2001	0.0623	0.9015	0.047*	
H21C	−0.0031	0.1045	0.9524	0.047*	

### Atomic displacement parameters (Å^2^)

**Table:**

	*U*^11^	*U*^22^	*U*^33^	*U*^12^	*U*^13^	*U*^23^
O1	0.0561 (19)	0.0329 (18)	0.0277 (15)	0.0003 (16)	0.0204 (14)	−0.0033 (15)
O2	0.0502 (18)	0.0226 (18)	0.0357 (17)	−0.0006 (14)	0.0189 (14)	0.0045 (14)
O3	0.0308 (14)	0.0276 (16)	0.0280 (14)	−0.0096 (14)	0.0108 (11)	−0.0054 (13)
O4	0.0270 (13)	0.0223 (15)	0.0321 (14)	0.0074 (12)	0.0132 (11)	0.0065 (13)
O5	0.0359 (15)	0.0235 (16)	0.0254 (14)	−0.0018 (13)	0.0099 (11)	0.0038 (13)
N1	0.0271 (16)	0.0230 (19)	0.0244 (17)	−0.0037 (14)	0.0065 (13)	−0.0036 (15)
N2	0.0203 (14)	0.0194 (17)	0.0210 (14)	−0.0040 (12)	0.0087 (11)	0.0000 (13)
N3	0.0208 (14)	0.0169 (16)	0.0221 (15)	0.0033 (13)	0.0043 (11)	0.0014 (13)
C1	0.0181 (16)	0.023 (2)	0.0190 (17)	−0.0018 (15)	0.0037 (13)	−0.0007 (16)
C2	0.0188 (16)	0.0200 (19)	0.0216 (17)	−0.0012 (15)	0.0027 (13)	−0.0014 (16)
C3	0.0216 (17)	0.021 (2)	0.0215 (18)	−0.0018 (15)	0.0030 (14)	−0.0031 (16)
C4	0.0231 (17)	0.027 (2)	0.0251 (18)	−0.0018 (16)	0.0046 (14)	−0.0064 (18)
C5	0.026 (2)	0.019 (2)	0.034 (2)	0.0037 (16)	0.0035 (16)	−0.0049 (17)
C6	0.0236 (18)	0.017 (2)	0.0263 (19)	−0.0004 (15)	0.0021 (14)	0.0023 (17)
C7	0.0186 (16)	0.023 (2)	0.0218 (17)	0.0017 (16)	0.0039 (13)	0.0038 (16)
C8	0.0151 (16)	0.021 (2)	0.0202 (17)	−0.0026 (14)	0.0022 (13)	−0.0001 (16)
C9	0.0188 (15)	0.0165 (19)	0.0213 (18)	−0.0012 (14)	0.0053 (13)	−0.0019 (16)
C10	0.0156 (15)	0.0189 (19)	0.0227 (18)	−0.0002 (15)	0.0030 (13)	−0.0011 (17)
C11	0.0197 (16)	0.021 (2)	0.0227 (17)	0.0001 (16)	0.0061 (13)	−0.0013 (17)
C12	0.0227 (17)	0.0156 (19)	0.030 (2)	0.0000 (15)	0.0027 (15)	−0.0003 (17)
C13	0.0207 (16)	0.019 (2)	0.0239 (18)	−0.0027 (15)	0.0038 (14)	0.0058 (16)
C14	0.0181 (16)	0.0194 (19)	0.0219 (18)	−0.0013 (15)	0.0018 (13)	−0.0022 (16)
C15	0.0164 (15)	0.020 (2)	0.0189 (17)	−0.0005 (14)	0.0023 (13)	−0.0014 (16)
C16	0.0226 (18)	0.019 (2)	0.0244 (19)	0.0015 (16)	0.0065 (15)	−0.0033 (17)
C17	0.0225 (17)	0.0146 (19)	0.0282 (19)	−0.0001 (15)	0.0045 (14)	0.0008 (17)
C18	0.0202 (17)	0.024 (2)	0.0211 (17)	−0.0045 (15)	0.0015 (14)	0.0017 (17)
C19	0.0290 (19)	0.024 (2)	0.0235 (18)	0.0011 (16)	0.0090 (15)	−0.0035 (17)
C20	0.0258 (18)	0.021 (2)	0.0245 (19)	0.0027 (16)	0.0061 (15)	0.0013 (17)
C21	0.031 (2)	0.030 (2)	0.033 (2)	−0.0023 (19)	0.0060 (17)	0.012 (2)

### Geometric parameters (Å, º)

**Table:**

O1—N1	1.235 (5)	C8—C9	1.394 (5)
O2—N1	1.216 (5)	C8—C13	1.401 (6)
O3—C7	1.221 (5)	C9—C10	1.390 (5)
O4—C14	1.229 (5)	C9—H9	0.9500
O5—C18	1.372 (5)	C10—C11	1.398 (6)
O5—C21	1.425 (5)	C10—C14	1.498 (5)
N1—C3	1.465 (5)	C11—C12	1.396 (6)
N2—C7	1.346 (5)	C11—H11	0.9500
N2—C8	1.425 (5)	C12—C13	1.388 (5)
N2—H2N	0.8800	C12—H12	0.9500
N3—C14	1.359 (5)	C13—H13	0.9500
N3—C15	1.424 (5)	C15—C16	1.391 (5)
N3—H3N	0.8800	C15—C20	1.396 (5)
C1—C2	1.388 (6)	C16—C17	1.388 (6)
C1—C6	1.389 (6)	C16—H16	0.9500
C1—C7	1.511 (5)	C17—C18	1.383 (6)
C2—C3	1.389 (5)	C17—H17	0.9500
C2—H2	0.9500	C18—C19	1.392 (6)
C3—C4	1.383 (6)	C19—C20	1.380 (6)
C4—C5	1.376 (6)	C19—H19	0.9500
C4—H4	0.9500	C20—H20	0.9500
C5—C6	1.389 (6)	C21—H21A	0.9800
C5—H5	0.9500	C21—H21B	0.9800
C6—H6	0.9500	C21—H21C	0.9800
			
C18—O5—C21	117.5 (3)	C9—C10—C14	116.1 (4)
O2—N1—O1	122.6 (4)	C11—C10—C14	123.2 (4)
O2—N1—C3	119.7 (3)	C12—C11—C10	119.2 (4)
O1—N1—C3	117.7 (4)	C12—C11—H11	120.4
C7—N2—C8	124.3 (3)	C10—C11—H11	120.4
C7—N2—H2N	117.8	C13—C12—C11	120.6 (4)
C8—N2—H2N	117.8	C13—C12—H12	119.7
C14—N3—C15	125.8 (3)	C11—C12—H12	119.7
C14—N3—H3N	117.1	C12—C13—C8	119.9 (4)
C15—N3—H3N	117.1	C12—C13—H13	120.0
C2—C1—C6	120.1 (4)	C8—C13—H13	120.0
C2—C1—C7	123.0 (4)	O4—C14—N3	123.0 (4)
C6—C1—C7	116.7 (4)	O4—C14—C10	121.4 (3)
C1—C2—C3	117.3 (4)	N3—C14—C10	115.5 (4)
C1—C2—H2	121.3	C16—C15—C20	119.2 (4)
C3—C2—H2	121.3	C16—C15—N3	123.4 (4)
C4—C3—C2	123.4 (4)	C20—C15—N3	117.4 (3)
C4—C3—N1	118.6 (4)	C17—C16—C15	119.7 (4)
C2—C3—N1	117.9 (4)	C17—C16—H16	120.2
C5—C4—C3	118.2 (4)	C15—C16—H16	120.2
C5—C4—H4	120.9	C18—C17—C16	121.0 (4)
C3—C4—H4	120.9	C18—C17—H17	119.5
C4—C5—C6	120.0 (4)	C16—C17—H17	119.5
C4—C5—H5	120.0	O5—C18—C17	124.9 (4)
C6—C5—H5	120.0	O5—C18—C19	115.6 (4)
C5—C6—C1	120.9 (4)	C17—C18—C19	119.4 (4)
C5—C6—H6	119.5	C20—C19—C18	119.9 (4)
C1—C6—H6	119.5	C20—C19—H19	120.0
O3—C7—N2	124.7 (4)	C18—C19—H19	120.0
O3—C7—C1	120.0 (4)	C19—C20—C15	120.8 (4)
N2—C7—C1	115.2 (3)	C19—C20—H20	119.6
C9—C8—C13	119.7 (3)	C15—C20—H20	119.6
C9—C8—N2	121.8 (4)	O5—C21—H21A	109.5
C13—C8—N2	118.4 (4)	O5—C21—H21B	109.5
C10—C9—C8	120.1 (4)	H21A—C21—H21B	109.5
C10—C9—H9	119.9	O5—C21—H21C	109.5
C8—C9—H9	119.9	H21A—C21—H21C	109.5
C9—C10—C11	120.5 (4)	H21B—C21—H21C	109.5
			
C6—C1—C2—C3	1.6 (5)	C9—C10—C11—C12	−0.6 (5)
C7—C1—C2—C3	−173.7 (3)	C14—C10—C11—C12	175.0 (3)
C1—C2—C3—C4	−2.2 (5)	C10—C11—C12—C13	0.7 (6)
C1—C2—C3—N1	175.9 (3)	C11—C12—C13—C8	0.5 (6)
O2—N1—C3—C4	−177.1 (4)	C9—C8—C13—C12	−2.0 (6)
O1—N1—C3—C4	3.1 (5)	N2—C8—C13—C12	−179.2 (3)
O2—N1—C3—C2	4.7 (5)	C15—N3—C14—O4	−0.2 (6)
O1—N1—C3—C2	−175.2 (3)	C15—N3—C14—C10	−178.0 (3)
C2—C3—C4—C5	0.0 (6)	C9—C10—C14—O4	28.3 (5)
N1—C3—C4—C5	−178.1 (3)	C11—C10—C14—O4	−147.5 (4)
C3—C4—C5—C6	2.8 (6)	C9—C10—C14—N3	−153.8 (3)
C4—C5—C6—C1	−3.3 (6)	C11—C10—C14—N3	30.4 (5)
C2—C1—C6—C5	1.1 (6)	C14—N3—C15—C16	−26.6 (6)
C7—C1—C6—C5	176.7 (3)	C14—N3—C15—C20	155.5 (4)
C8—N2—C7—O3	1.3 (6)	C20—C15—C16—C17	−1.4 (6)
C8—N2—C7—C1	−178.4 (3)	N3—C15—C16—C17	−179.3 (3)
C2—C1—C7—O3	142.4 (4)	C15—C16—C17—C18	0.7 (6)
C6—C1—C7—O3	−33.1 (5)	C21—O5—C18—C17	1.8 (6)
C2—C1—C7—N2	−37.9 (5)	C21—O5—C18—C19	180.0 (4)
C6—C1—C7—N2	146.6 (3)	C16—C17—C18—O5	179.4 (3)
C7—N2—C8—C9	36.2 (5)	C16—C17—C18—C19	1.3 (6)
C7—N2—C8—C13	−146.6 (4)	O5—C18—C19—C20	179.3 (3)
C13—C8—C9—C10	2.1 (5)	C17—C18—C19—C20	−2.4 (6)
N2—C8—C9—C10	179.3 (3)	C18—C19—C20—C15	1.7 (6)
C8—C9—C10—C11	−0.9 (5)	C16—C15—C20—C19	0.3 (6)
C8—C9—C10—C14	−176.8 (3)	N3—C15—C20—C19	178.3 (4)

### Hydrogen-bond geometry (Å, º)

**Table:**

*D*—H···*A*	*D*—H	H···*A*	*D*···*A*	*D*—H···*A*
N2—H2*N*···O4^i^	0.88	2.09	2.908 (4)	155
N3—H3*N*···O3^ii^	0.88	2.21	3.056 (4)	163
C5—H5···O2^iii^	0.95	2.28	3.093 (5)	143
C19—H19···O1^iv^	0.95	2.44	3.365 (5)	164
C21—H21*C*···O1^v^	0.98	2.64	3.490 (5)	145

Symmetry codes: (i) −*x*+1, *y*+1/2, −*z*+1; (ii) −*x*, *y*+1/2, −*z*+1; (iii) *x*, *y*−1, *z*; (iv) *x*−1, *y*, *z*+1; (v) −*x*+1, *y*−1/2, −*z*+1.
