# Supplementary material for: Synthesis, crystal structure, Hirshfeld surface analysis, MEP study and mol­ecular docking of N-{3-[(4-meth­oxy­phen­yl)carbamo­yl]phen­yl}-3-nitro­benzamide as a promising inhibitor of hfXa
Source: Acta Crystallogr E Crystallogr Commun. 2020 Oct 20;76(Pt 11):1762–7. doi: 10.1107/S2056989020013730 (PMC7643242; doi:10.1107/S2056989020013730)
Supplement: Supplementary file 3 [file e-76-01762-sup3.pdf]

Supplementary information

Synthesis, crystal structure, Hirshfeld surface analysis, study MEP and molecular docking of N-(3-((4-methoxyphenyl)carbamoyl)phenyl)-3-nitrobenzamide as a promising inhibitor of hfXa.

Rodolfo Moreno-Fuquen,[a]* Mario Hurtado-Angulo,[a] Kevin Arango-Daraviña.[a] Gavin Bain,[b] and Alan R. Kennedy.[b]

[a]Grupo de Cristalografía. Departmento de Química, Universidad del Valle, A.A. 25360, Santiago de Cali, Colombia.

[b]WestCHEM. Department of Pure and Applied Chemistry, University of Strathclyde, 295 Cathedral Street, Glasgow G1 1XL, Scotland.

*Correspondence e-mail: rodolfo.moreno@correounivalle.edu,co

Synthesis and characterization.

General Information

All reagents were purchased from commercial sources and used without further purification. All starting materials were weighed and handled at room temperature. The reactions were controlled by thin layer chromatography (TLC) displayed by UV lamp (254 nm or 365 nm). Melting points were determined using a capillary melting point apparatus, SMP10 Stuart Fusiometer. The IR spectra were recorded in an FT-IR spectrophotometer with attenuated total reflection (ATR), IR-TF Shimadzu IRAffinity-1 and processed with the OriginPro 8 software. The mass spectra (MS) were recorded using a GC mass spectrometer with electronic impact ionization (IE) and electron multiplier detector, Shimadzu GCMS-QP2010. Readings in UV-Vis spectroscopy were made with Dimethylformamide (DMF) as solvent at room temperature.

Synthesis of N-(4-methoxyphenyl)-3-nitrobenzamide, (Ib), (Scheme 2).

The reaction was carried out between 4-methoxyaniline (50.3 mg, 0.406 mmol) and 3-nitrobenzoyl chloride, (b), (100.2 mg, 0.539 mmol), its subsequent purification allowed to obtain the compound (Ib), a greenish-yellow solid [Yield 91.8 mg (0.337 mmol), 83.0 %; p.f. 431-433 K]. FT-IR (ATR): υ (cm^{-1}) = 3293 (N-H), 3083, 2838, 1645 (C=O), 1614 (C=C), 1523, 1510, 1412, 1348 (NO_2), 1268, 1241, 1112, 1027, 912, 873, 827, 819, 769, 711, 668. MS (IE, 40 eV), m/z [M+] calculated for $C_{14}H_{12}N_2O_4$+: 272.08; found: 271.90

Synthesis of 3-amino-N-(4-methoxyphenyl)benzamide, (IIb), (Esquema 1).

The preparation and extraction of compound (IIb) is performed, using compound (Ib) as the starting reagent (90.1 mg, 0.331 mmol), [Yield 68.8 mg (0.284 mmol), 85.8 %; p.f. 396(1) K]. FT-IR (ATR): υ (cm^{-1})= 3441 and 3350 (NH_2), 3299 (N-H), 2959, 1625 (C=O), 1597 (C=C), 1578, 1515, 1489, 1413, 1320, 1269, 1240, 1174, 1111, 1027, 871, 810, 756, 686 cm-1. MS (IE, 40 eV), m/z [M+] calculated for $C_{14}H_{14}N_2O_2$+: 242.11; found: 242.05

Scheme 1. Synthetic route for obtaining N-(3-((4-methoxyphenyl)carbamoyl)phenyl)-3-nitrobenzamide, compound **I.**

Figures S1-S7 show the mass spectra (MS), infrared (IR) and visible ultraviolet (UV-Vis) of the different compounds synthesized in this work.

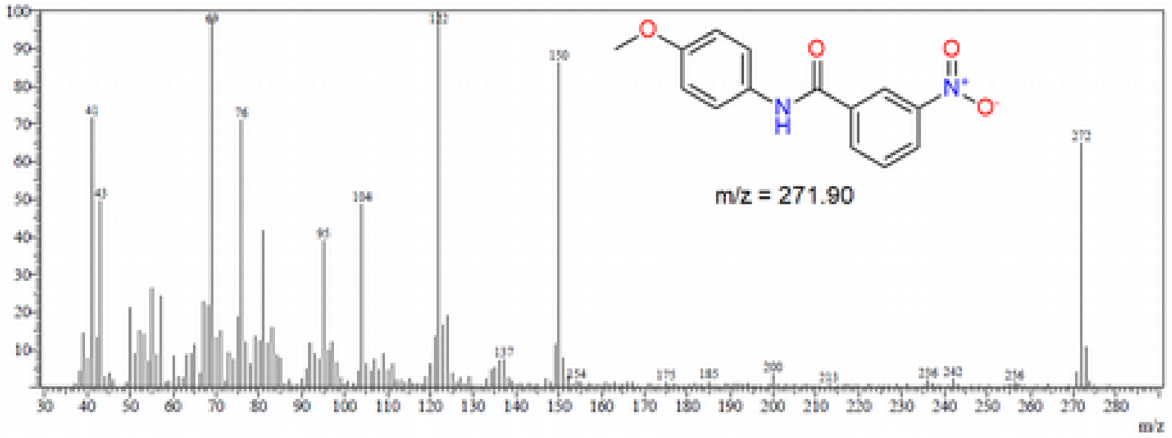

Figura S1 Mass spectrum of N-(4-methoxyphenyl)-3-nitrobenzamide, compound (I)

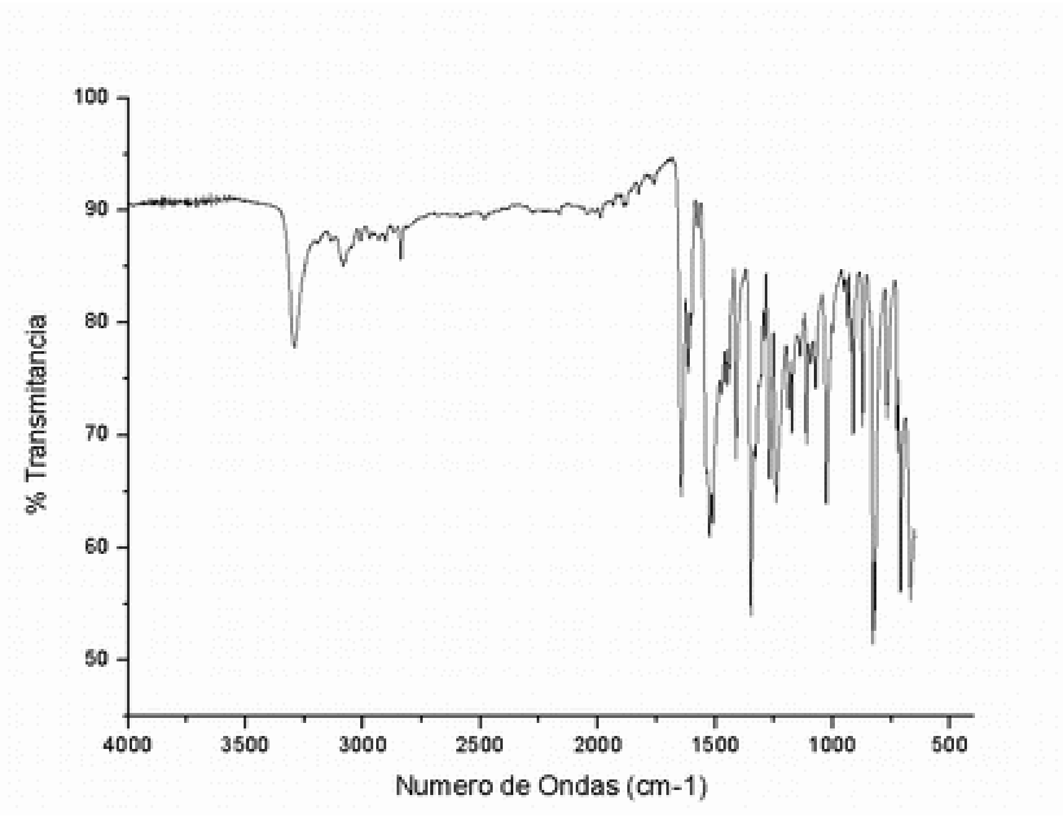

Figure S2. Infrared spectrum of 3-amino-N-(4-methoxyphenyl)benzamide, compound (I)

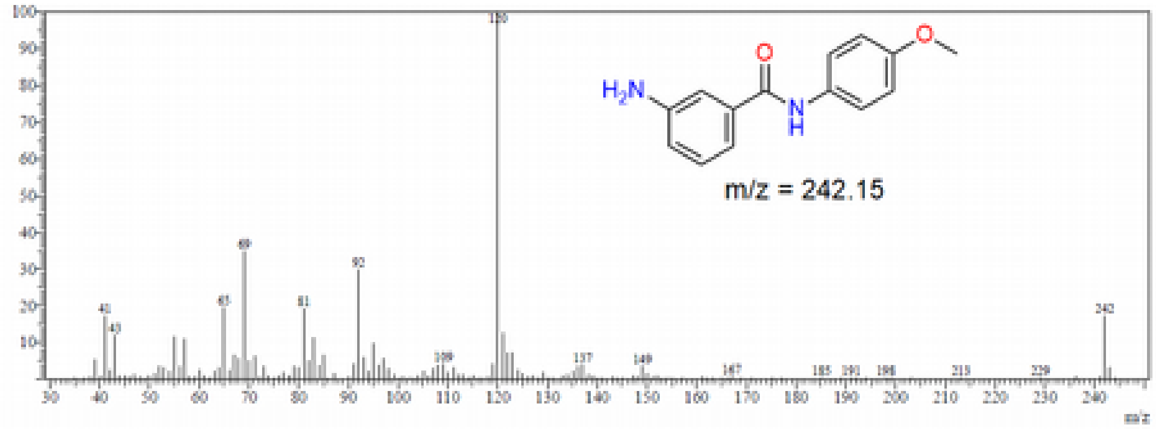

Figure S3. Mass spectrum for the compound (**II**)

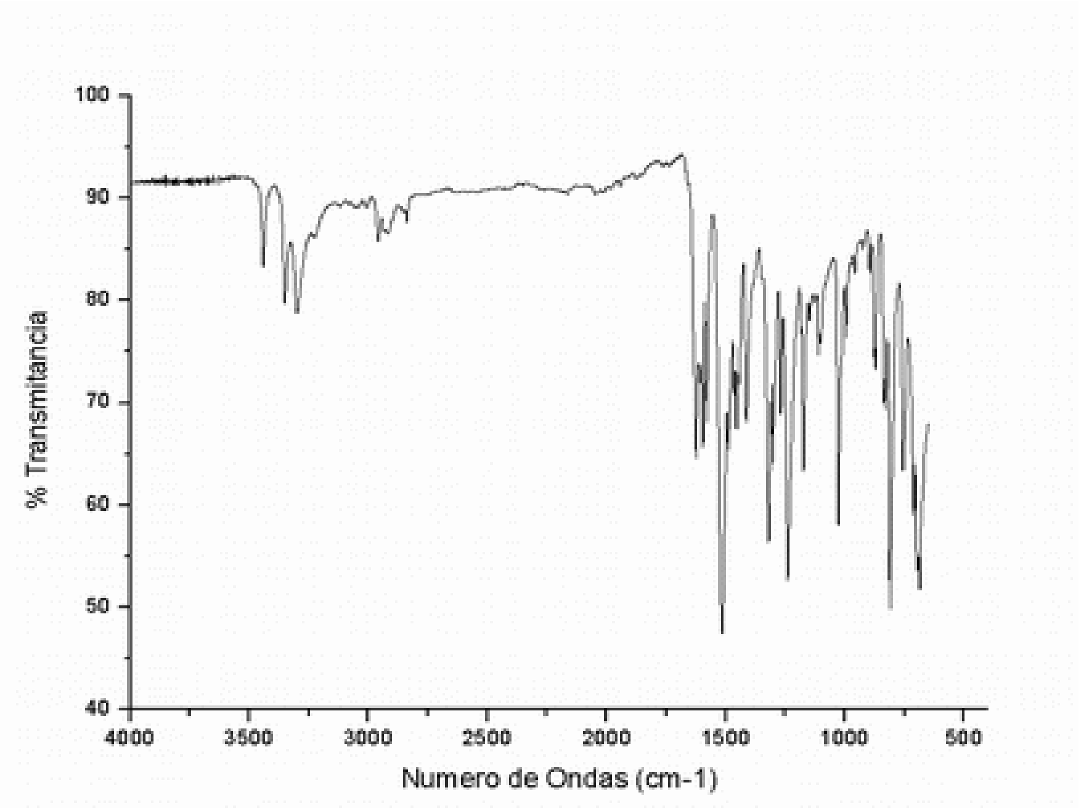

Figure S4. Infrared spectrum of the compound (II)

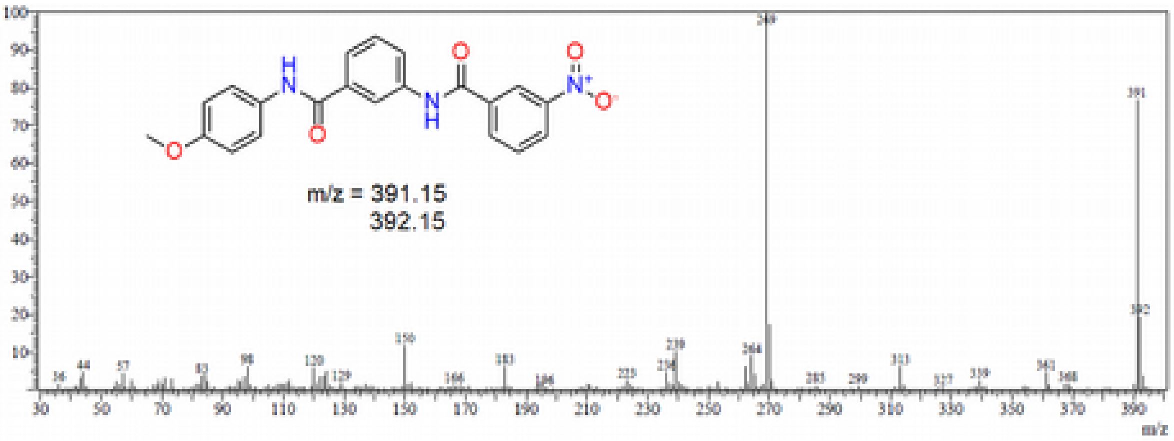

Figure S5. Mass spectrum for N-(3-((4-methoxyphenyl)carbamoyl)phenyl)-3 nitrobenzamide, compound (**I**)

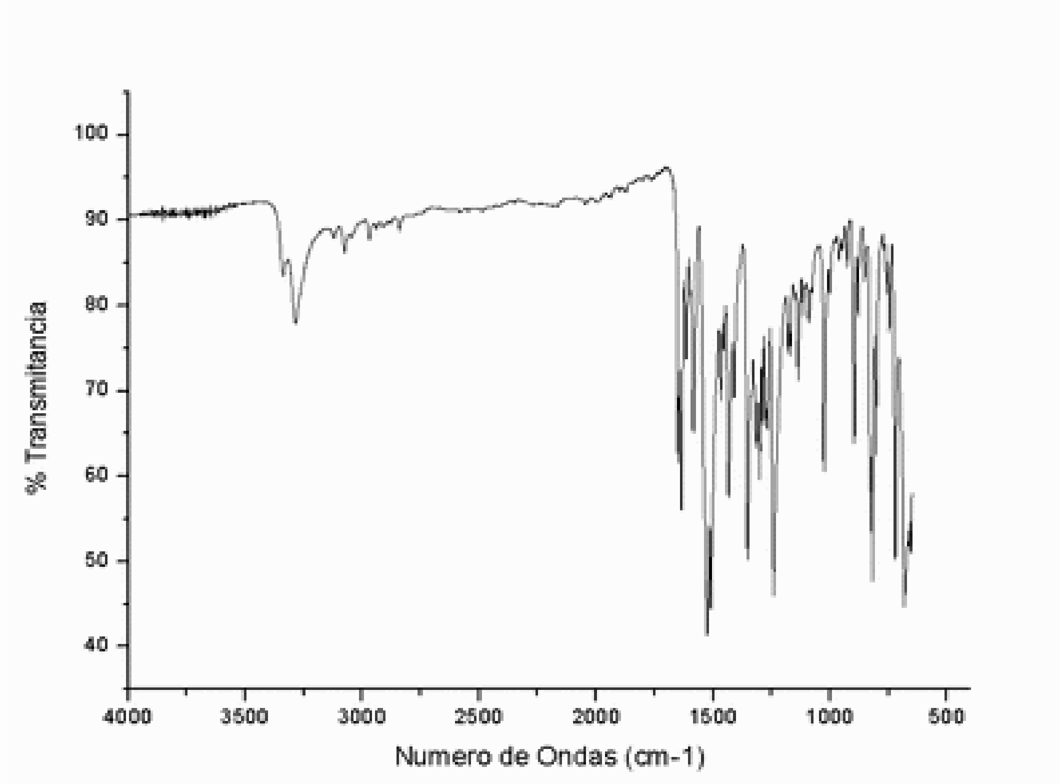

Figure S6. Infrared spectrum of the compound (**I**)

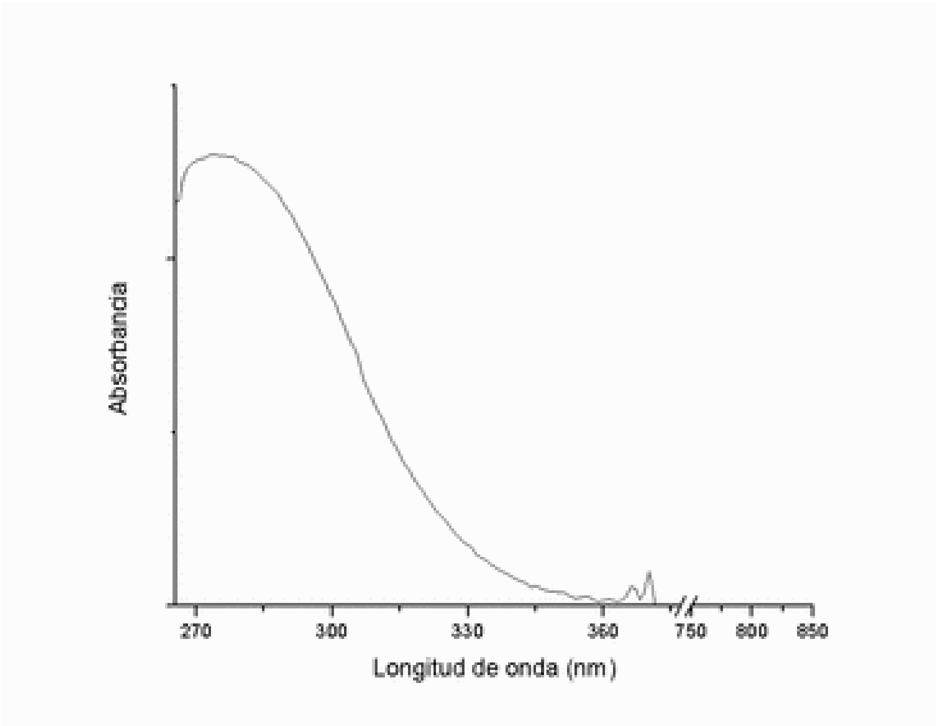

Figure S7. Espectro UV-vis del compuesto (**I**)

Table S1. Geometric parameters for (**I**) (Å, º)

O1—N1	1.235 (5)	C8—C9	1.394 (5)
O2—N1	1.216 (5)	C8—C13	1.401 (6)
O3—C7	1.221 (5)	C9—C10	1.390 (5)
O4—C14	1.229 (5)	C9—H9	0.9500
O5—C18	1.372 (5)	C10—C11	1.398 (6)
O5—C21	1.425 (5)	C10—C14	1.498 (5)
N1—C3	1.465 (5)	C11—C12	1.396 (6)
N2—C7	1.346 (5)	C11—H11	0.9500
N2—C8	1.425 (5)	C12—C13	1.388 (5)
N2—H1N	0.8800	C12—H12	0.9500
N3—C14	1.359 (5)	C13—H13	0.9500
N3—C15	1.424 (5)	C15—C16	1.391 (5)
N3—H2N	0.8800	C15—C20	1.396 (5)
C1—C2	1.388 (6)	C16—C17	1.388 (6)
C1—C6	1.389 (6)	C16—H16	0.9500
C1—C7	1.511 (5)	C17—C18	1.383 (6)
C2—C3	1.389 (5)	C17—H17	0.9500
C2—H2	0.9500	C18—C19	1.392 (6)
C3—C4	1.383 (6)	C19—C20	1.380 (6)
C4—C5	1.376 (6)	C19—H19	0.9500
C4—H4	0.9500	C20—H20	0.9500
C5—C6	1.389 (6)	C21—H21A	0.9800

C5—H5	0.9500	C21—H21B	0.9800
C6—H6	0.9500	C21—H21C	0.9800

C18—O5—C21	117.5 (3)	C9—C10—C14	116.1 (4)
O2—N1—O1	122.6 (4)	C11—C10—C14	123.2 (4)
O2—N1—C3	119.7 (3)	C12—C11—C10	119.2 (4)
O1—N1—C3	117.7 (4)	C12—C11—H11	120.4
C7—N2—C8	124.3 (3)	C10—C11—H11	120.4
C7—N2—H1N	117.8	C13—C12—C11	120.6 (4)
C8—N2—H1N	117.8	C13—C12—H12	119.7
C14—N3—C15	125.8 (3)	C11—C12—H12	119.7
C14—N3—H2N	117.1	C12—C13—C8	119.9 (4)
C15—N3—H2N	117.1	C12—C13—H13	120.0
C2—C1—C6	120.1 (4)	C8—C13—H13	120.0
C2—C1—C7	123.0 (4)	O4—C14—N3	123.0 (4)
C6—C1—C7	116.7 (4)	O4—C14—C10	121.4 (3)
C1—C2—C3	117.3 (4)	N3—C14—C10	115.5 (4)
C1—C2—H2	121.3	C16—C15—C20	119.2 (4)
C3—C2—H2	121.3	C16—C15—N3	123.4 (4)
C4—C3—C2	123.4 (4)	C20—C15—N3	117.4 (3)
C4—C3—N1	118.6 (4)	C17—C16—C15	119.7 (4)
C2—C3—N1	117.9 (4)	C17—C16—H16	120.2
C5—C4—C3	118.2 (4)	C15—C16—H16	120.2
C5—C4—H4	120.9	C18—C17—C16	121.0 (4)
C3—C4—H4	120.9	C18—C17—H17	119.5
C4—C5—C6	120.0 (4)	C16—C17—H17	119.5
C4—C5—H5	120.0	O5—C18—C17	124.9 (4)
C6—C5—H5	120.0	O5—C18—C19	115.6 (4)
C5—C6—C1	120.9 (4)	C17—C18—C19	119.4 (4)
C5—C6—H6	119.5	C20—C19—C18	119.9 (4)
C1—C6—H6	119.5	C20—C19—H19	120.0
O3—C7—N2	124.7 (4)	C18—C19—H19	120.0
O3—C7—C1	120.0 (4)	C19—C20—C15	120.8 (4)
N2—C7—C1	115.2 (3)	C19—C20—H20	119.6
C9—C8—C13	119.7 (3)	C15—C20—H20	119.6
C9—C8—N2	121.8 (4)	O5—C21—H21A	109.5
C13—C8—N2	118.4 (4)	O5—C21—H21B	109.5
C10—C9—C8	120.1 (4)	H21A—C21—H21B	109.5
C10—C9—H9	119.9	O5—C21—H21C	109.5
C8—C9—H9	119.9	H21A—C21—H21C	109.5
C9—C10—C11	120.5 (4)	H21B—C21—H21C	109.5

C6—C1—C2—C3	1.6 (5)	C9—C10—C11—C12	−0.6 (5)
C7—C1—C2—C3	−173.7 (3)	C14—C10—C11—C12	175.0 (3)
C1—C2—C3—C4	−2.2 (5)	C10—C11—C12—C13	0.7 (6)
C1—C2—C3—N1	175.9 (3)	C11—C12—C13—C8	0.5 (6)
O2—N1—C3—C4	−177.1 (4)	C9—C8—C13—C12	−2.0 (6)
O1—N1—C3—C4	3.1 (5)	N2—C8—C13—C12	−179.2 (3)

O2—N1—C3—C2	4.7 (5)	C15—N3—C14—O4	−0.2 (6)
O1—N1—C3—C2	−175.2 (3)	C15—N3—C14—C10	−178.0 (3)
C2—C3—C4—C5	0.0 (6)	C9—C10—C14—O4	28.3 (5)
N1—C3—C4—C5	−178.1 (3)	C11—C10—C14—O4	−147.5 (4)
C3—C4—C5—C6	2.8 (6)	C9—C10—C14—N3	−153.8 (3)
C4—C5—C6—C1	−3.3 (6)	C11—C10—C14—N3	30.4 (5)
C2—C1—C6—C5	1.1 (6)	C14—N3—C15—C16	−26.6 (6)
C7—C1—C6—C5	176.7 (3)	C14—N3—C15—C20	155.5 (4)
C8—N2—C7—O3	1.3 (6)	C20—C15—C16—C17	−1.4 (6)
C8—N2—C7—C1	−178.4 (3)	N3—C15—C16—C17	−179.3 (3)
C2—C1—C7—O3	142.4 (4)	C15—C16—C17—C18	0.7 (6)
C6—C1—C7—O3	−33.1 (5)	C21—O5—C18—C17	1.8 (6)
C2—C1—C7—N2	−37.9 (5)	C21—O5—C18—C19	180.0 (4)
C6—C1—C7—N2	146.6 (3)	C16—C17—C18—O5	179.4 (3)
C7—N2—C8—C9	36.2 (5)	C16—C17—C18—C19	1.3 (6)
C7—N2—C8—C13	−146.6 (4)	O5—C18—C19—C20	179.3 (3)
C13—C8—C9—C10	2.1 (5)	C17—C18—C19—C20	−2.4 (6)
N2—C8—C9—C10	179.3 (3)	C18—C19—C20—C15	1.7 (6)
C8—C9—C10—C11	−0.9 (5)	C16—C15—C20—C19	0.3 (6)
C8—C9—C10—C14	−176.8 (3)	N3—C15—C20—C19	178.3 (4)

Structural Features.

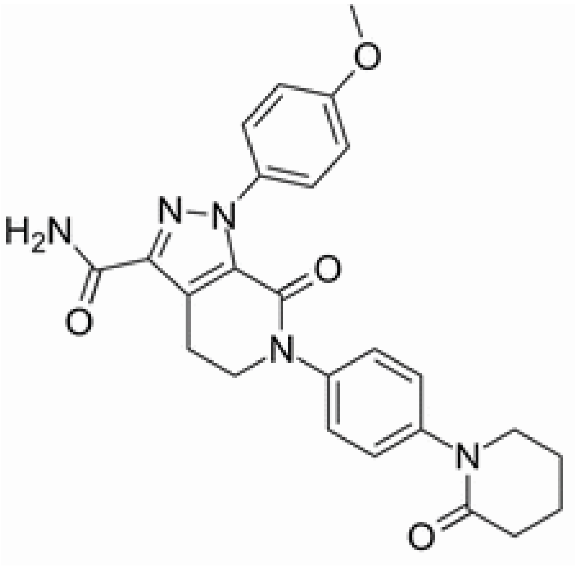

Figure S8. M**olecular diagram of Apixaban**

Docking Molecular.

Tables S2-S3 and Figure S18 show the complementary results obtained from Molecular Coupling calculations for the control molecule Apixaban and compounds (I).

Working box parameters for molecular coupling calculations in AutoDock vina:

center_x = 7.972
center_y = 42.838
center_z = 61.911

size_x = 14
size_y = 14
size_z = 16

Table S2. Results Molecular coupling for the Apixaban ligand.

Mode	***Affinity (kcal/mol)***	***Dist from RMSD l.b.***	***Best mode RMSD u.b.***
1	-10.7	0	0
2	-8.1	1.601	2.001

Table S3. Results of Molecular Coupling Ligand Compound I calculations

**Mode**	**Affinity (kcal/mol)**	**Dist from RMSD l.b.**	**best mode RMSD u.b.**
1	-7.7	0	0
2	-7.6	4.306	9.223
3	-7.4	3.090	9.103
4	-7.3	4.718	5.209
5	-7.1	5.559	7.413
6	-7.0	4.313	9.598
7	-6.9	5.008	9.566
8	-6.6	3.326	3.879
9	-6.6	5.249	7.457

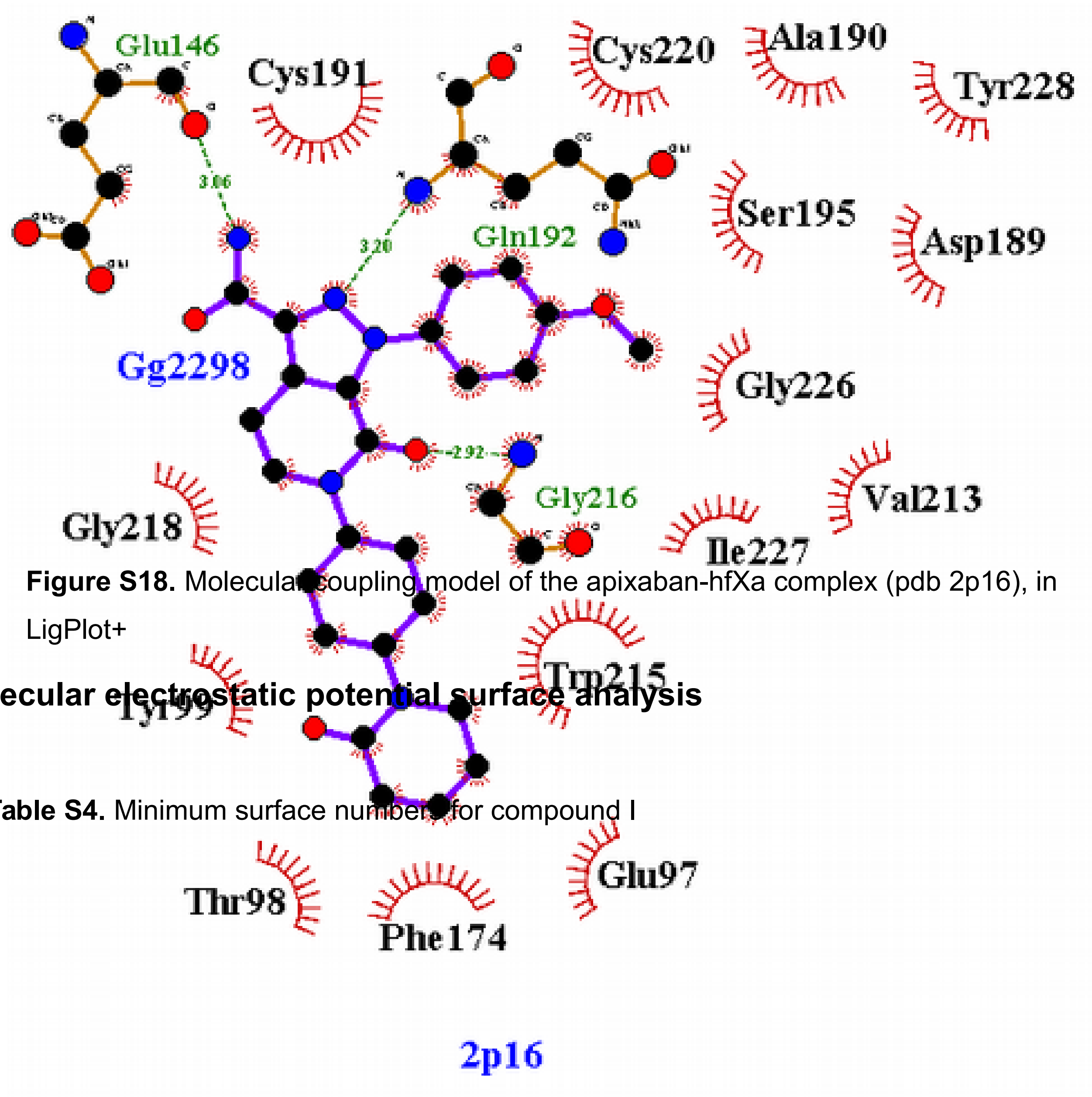

Figure S18. Molecular coupling model of the apixaban-hfXa complex (pdb 2p16), in LigPlot+

Molecular electrostatic potential surface analysis

Table S4. Minimum surface numbers for compound I

#	a.u.	eV	kcal/mol	X/Y/Z coordinate(Angstrom		
1	-0.04516233	-1.22893	-28.339814	-9.868389	1.394945	1.425754
2	-0.04441575	-1.208614	-27.871329	-8.560326	2.577916	1.662683
3	0.0073569	0.200191	4.616525	-5.904203	-1.908719	-2.247358
4	-0.05518866	-1.50176	-34.631439	-0.778451	-2.803837	-0.296534
5	-0.00766359	-0.208537	-4.808979	-0.245094	2.718346	1.264674
* 6	-0.06728349	-1.830877	-42.221066	1.450564	-2.178549	-1.519763
7	-0.02738431	-0.745165	-17.18393	5.250137	-1.493272	1.673143
8	-0.02453883	-0.667736	-15.398362	5.979693	-0.188778	2.334671
9	-0.02601508	-0.707906	-16.324725	6.349401	-0.056599	-1.811335
10	-0.02373462	-0.645852	-14.893709	6.842408	1.243444	-1.105037
11	0.0196632	0.535063	12.338856	8.419975	-3.701722	-0.516742
12	-0.03691966	-1.004635	-23.167458	9.922357	0.201005	1.65882
13	-0.03691541	-1.004519	-23.164786	9.964256	0.346853	1.412524

Table S5. Maximum area numbers for the compound (I)

#	a.u.	eV	kcal/mol	X/Y/Z coordinate(Angstrom		
1	0.03640107	0.990524	22.842038	-7.97481	-3.559718	-0.341963

2	0.03056962	0.831842	19.182744	-7.585801	0.79812	-0.972743
3	0.02803824	0.762959	17.594279	-7.061884	-0.265495	2.390307
4	0.0385276	1.048389	24.176452	-6.925404	-4.89161	-0.945267
5	0.01546713	0.420882	9.705778	-5.935384	-0.721873	-1.64844
6	0.01065223	0.289862	6.684378	-5.545125	-1.787171	1.644195
* 7	0.08124016	2.210657	50.979012	-4.086395	2.225001	-0.558586
8	-0.00566683	-0.154202	-3.55599	0.015829	2.055995	1.126935
9	0.03299604	0.897868	20.705348	0.063493	5.681633	-0.940929
10	0.06840122	1.861292	42.922452	3.472597	2.993343	0.885372
11	-0.02265577	-0.616495	-14.216725	6.658461	0.621018	-1.204767
12	0.02399621	0.65297	15.057859	7.448472	-3.503854	-0.711713
13	0.01793313	0.487985	11.253221	8.262496	2.634639	1.958306
14	0.02107523	0.573486	13.224919	8.425487	-4.225388	0.879377
15	0.0213208	0.580168	13.379013	9.119077	-3.108161	-1.822352
16	0.02422566	0.659214	15.201846	9.94839	-3.680203	-0.201481
17	0.02443546	0.664923	15.333493	11.529277	-2.687265	0.754257

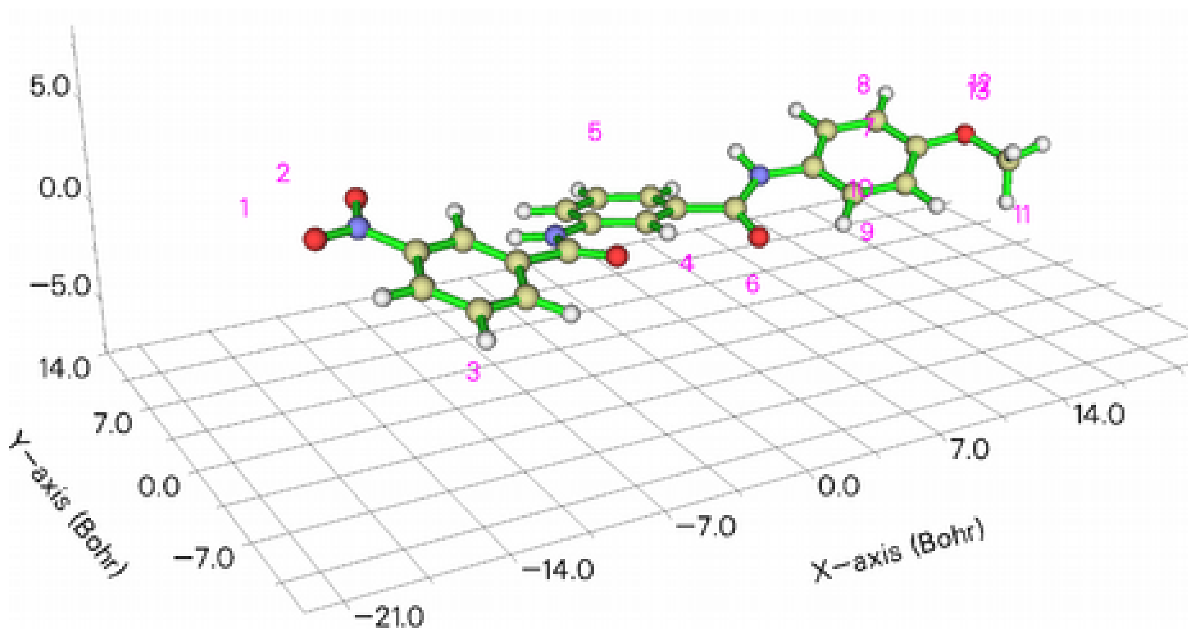

Figure S19. Minimum surface numbers (pink) on composite structure I

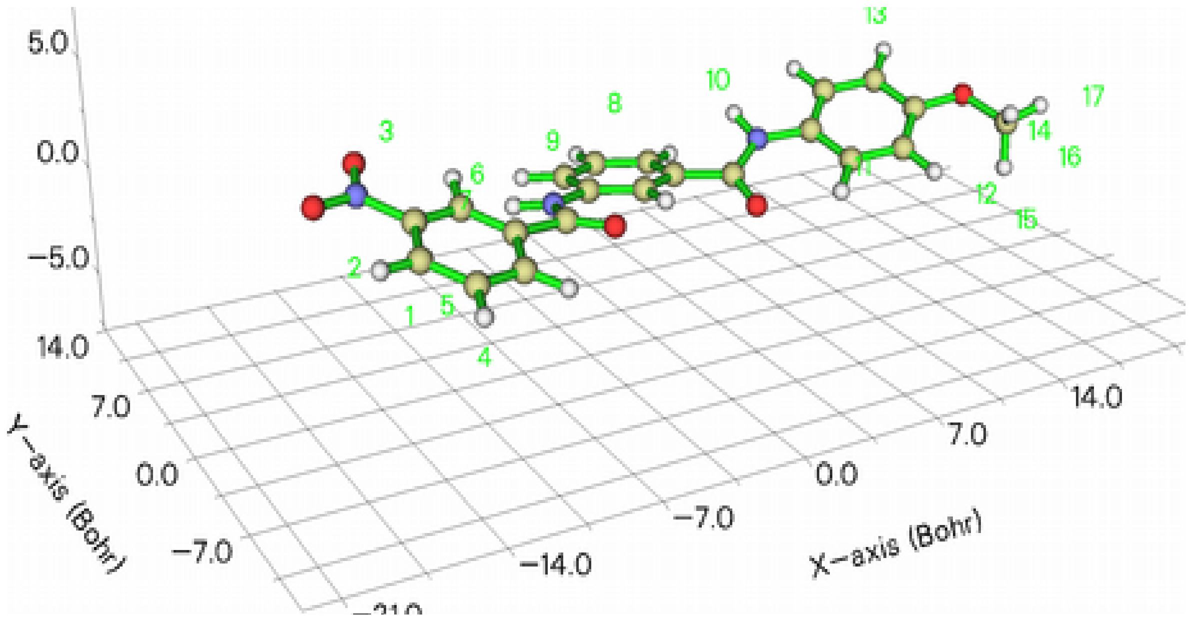

Figure 20. Maximum surface numbers (green) on composite structure I
